# The yield of mechanically harvested rapeseed (*Brassica napus* L.) can be increased by optimum plant density and row spacing

**DOI:** 10.1038/srep18835

**Published:** 2015-12-21

**Authors:** Jie Kuai, Yingying Sun, Qingsong Zuo, Haidong Huang, Qingxi Liao, Chongyou Wu, Jianwei Lu, Jiangsheng Wu, Guangsheng Zhou

**Affiliations:** 1 College of Plant Science and Technology, Huazhong Agricultural University, Wuhan 430070, Hubei Province, P.R. China; 2 Key Laboratory of Crop Genetics and Physiology of Jiangsu Province, Yangzhou University, Yangzhou 225009, Jiangsu Province, P.R. China; 3 Institute of agricultural mechanization ministry of agriculture, Nanjing 210014, Jiangsu Province, P.R. China

## Abstract

To determine the effects of plant density and row spacing on the mechanical harvesting of rapeseed (*Brassica napus* L.), field experiments were conducted. Higher plant density produced fewer pods and reduced the yield per plant. Wider row spacing at higher plant densities increased seeds per pod and the 1000-seed weight, resulting in a higher yield per plant. The highest yields were achieved at a density of 45 × 10^4^ plants ha^−1^ (D45) in combination with 15 cm row spacing (R15) because mortality associated with competition increased as both the plant density and row spacing increased. The leaf area index (LAI) and pod area index (PAI) showed similar relations to the yield per hectare, and they were positively correlated with the percentage of intercepted light, whereas the radiation use efficiency (RUE) was positively correlated with population biomass. Reduced plant height and increased root/shoot ratios led to a decreased culm lodging index. Improved resistance to pod shattering was also observed as plant density and row spacing increased. The angle of the lowest 5 branches decreased as row spacing increased under D30 and D45. All of these structural changes influenced the mechanical harvesting operations, resulting in the highest yield of mechanically harvesting rapeseed under D45R15.

Conventional rapeseed (*Brassica napus* L.) production in China has been dependent on manual practices for decades. However, hand-harvesting of rapeseed is labor-intensive, and the supply of dependable, skilled labor is of concern to rapeseed producers in China because the proportion of domestic labor resources engaged in crop farming is sharply decreasing^1^. Mechanization is an important and efficient tool for enhancing crop yield; it also reduces the labor associated with crop production and ultimately increases farmers’ prosperity^2^. In fact, mechanized direct-seeding has increasingly been practiced in the major canola-producing regions of China, but the development of mechanized harvesting has lagged behind. Currently, the lower rate of mechanization and the greater emphasis on human labor for harvesting are major factors restricting rapeseed production in China^3^. China will not remain competitive in the world market for rapeseed with the current lack of research on mechanical harvesting. Therefore, there is an immediate need to develop a mechanized harvesting system for rapeseed production.

Rapeseed yield and mechanical harvesting efficiency have been widely affected by agronomic practices, among which, plant density and row spacing have played a vital role in obtaining higher yields^4^. Thus, it is important to determine the appropriate plant density and row spacing that optimize both the seed yield and mechanical harvesting efficiency. Numerous researchers have investigated the effects of plant density and row spacing on agronomic traits and the yield of rapeseed. Research to determine the optimal plant density in combination with row spacing for the maximum mechanized production of rapeseed has been inconclusive because the results vary depending on the location, cultivar, soil type and local climate.

Rapeseed growth, yield and yield components are directly affected by plant density^5^. In general, plant densities of 60–70 plants m^−2^ are considered to be optimal^6^ for rapeseed hybrids in Europe, whereas the typical plant density of hybrid rapeseed in China is approximately 30 plants m^−2^ 7,8,9. As plant densities decline, the reduction in the number of plants per unit area is partially compensated for by an accompanying increase in the productivity of each plant, as a result of greater leaf area; more branches; and a greater number of pods per plant^10^. The rapeseed yield typically exhibits a quadratic response to plant density, with a near-linear increase across a range of low densities, a gradual decrease in the rate of yield increase, and finally, a maximum yield at the optimum plant density, which depends upon crop species, environmental conditions and agronomic factors^5^,^7^,^10^. Leach *et al.* (1999) found the maximum seed yield of winter rapeseed occurred at a density of 50–60 plants m^−2^ in a series of multi-factorial experiments^10^, whereas a field experiment by Momoh and Zhou (2001) found that the highest seed yield of transplanted winter rapeseed occurred at plant densities of 9.75  ×  10^4^ and 12.75 × 10^4^ plant ha^−1^ 7. Other studies have reported that different seeding rates (7–14 kg ha^−1^) had no significant effect on seed yield^11^.

Compared with conventional wide row spacing, rapeseed produced in narrow rows generally has superior yields when conditions are favorable. Narrow rows increase the total seasonal light interception, make more efficient use of available light and should allow for more rapid canopy closure and shading of the ground, thereby improving weed control^12^. Christensen and Drabble reported that both *Brassica napus* and *Brassica rapa* showed a higher grain yield with narrower row spacing (7.5 cm) compared with wider spacing (15 and 23 cm)^11^. Within a certain range, the yield of winter rapeseed increased with narrower row spacing from 30 to 15 cm or from 35 to 17.5 cm^13^,^14^. Similar results were obtained with cotton (*Gossypium hirsutum* L.)^15^, soybeans (*Glycine max* L.)^16^,^17^ and corn (*Zea mays* L.)^12^,^18^. However, the rapeseed yield will decrease when the row spacing is set much narrower than 15 cm. Shanhin and Valiollah (2009) showed that the seed yield of winter rapeseed grown at a 12 cm row spacing was lower than winter rapeseed grown at 24 cm. Moreover, mechanical cultivation, fertilizer application and post-emergence herbicide treatments are more difficult in narrow row cultivation^19^.

Rapeseed with greater resistance to lodging and pod shattering would permit the harvester to operate more efficiently. Lodging and pod-shatter resistance could be improved with appropriate plant densities and row spacing. At too high a density, rapeseed is often more susceptible to lodging^10^. However, Robinson (1986) noted more lodging at lower densities because of larger, heavier stems^20^. In general, higher plant densities are more suitable for the mechanical harvesting of grain amaranth compared to lower densities. This is because higher densities promote less branching, fewer secondary seed heads, smaller stalk diameters, and more uniform maturation^21^. However, high population densities may be less suitable in more arid climates due to greater competition for available soil moisture^22^. Lodging increased significantly with an increase in plant density at both 1.0 m and 0.5 m row spacings in soybeans (*Glycine max* L.), and more severe stem lodging and higher plant mortality were observed for high plant densities at the 1.0 m row spacing compared with the 0.5 m row spacing^16^, whereas other results have reported that lodging was reduced by increased row spacing in high-yielding irrigated wheat^23^. The use of narrow rows resulted in lower plant mortality and associated stand losses during the growing season and less plant lodging in grain amaranth (*Amaranthus spp.*)^18^. Information on changes in the pattern of pod shattering under different plant densities and row spacings, which are critical to the mechanical harvesting of rapeseed, is very limited.

There have been few published studies on the response of mechanical harvesting traits in rapeseed to changes in plant density and row spacing. Information on plant density, row spacing, and their possible interaction has not been well described. In addition, inconsistent results from previously published studies suggest that further research is needed. Growing rapeseed in alternating rows with different plant densities has the potential to increase seed yield and promote the mechanical harvesting efficiency. Consequently, the objective of this study was to determine the effects of plant density and row spacing on the rapeseed yield and mechanical harvesting properties. A balance was sought between conditions that facilitated mechanical harvesting, but did not adversely affect the yield of rapeseed by altering the plant density and row spacing required for maximizing yield.

## Results

### Weather data

The amount of monthly sunshine and the cumulative temperature of both the seedling to wintering stage and the flowering to pod-filling stage were higher in 2013–2014 than in 2012–2013, but monthly precipitation was lower in 2013–2014. A similar trend in monthly sunshine, cumulative temperature and monthly precipitation was observed over the entire growth period (Table 1).

### Yield and yield components

The mature plant rate was significantly affected by plant density and row spacing. In general, the mature plant rate decreased with increasing plant density and row spacing; the highest mature plant rate was observed under D15R25, where the plant spacing was 26.7 cm. At the same plant density, a smaller difference between row spacing and plant spacing produced a higher plant maturity rate (Table 2).

The seed yield and yield components varied significantly with changes in plant density and row spacing. Under the same row spacing, the number of pods per plant, seeds per pod, 1000-seed weight and seed yield per plant all decreased with increasing plant density, whereas the seed yield per hectare increased. In contrast, under the same plant density (D30 and D45), the number of seeds per pod, the 1000-seed weight and the seed yield per plant increased with increasing row spacing, whereas the seed yield per hectare significantly decreased. Among all of the treatments, D45R15 consistently produced the highest seed yield, while D15R15 had the lowest seed yield in both growing seasons. Population biomass showed the same trend as seed yield. Increasing plant density significantly decreased the biomass per plant, whereas plant spacing had a smaller effect on biomass, particularly under higher plant densities (D30 and D45). Significant interactions between row spacing and plant density were found for seed yield and yield components (Table 3).

### Leaf area index, pod area index and harvest index

**Figure 1:**
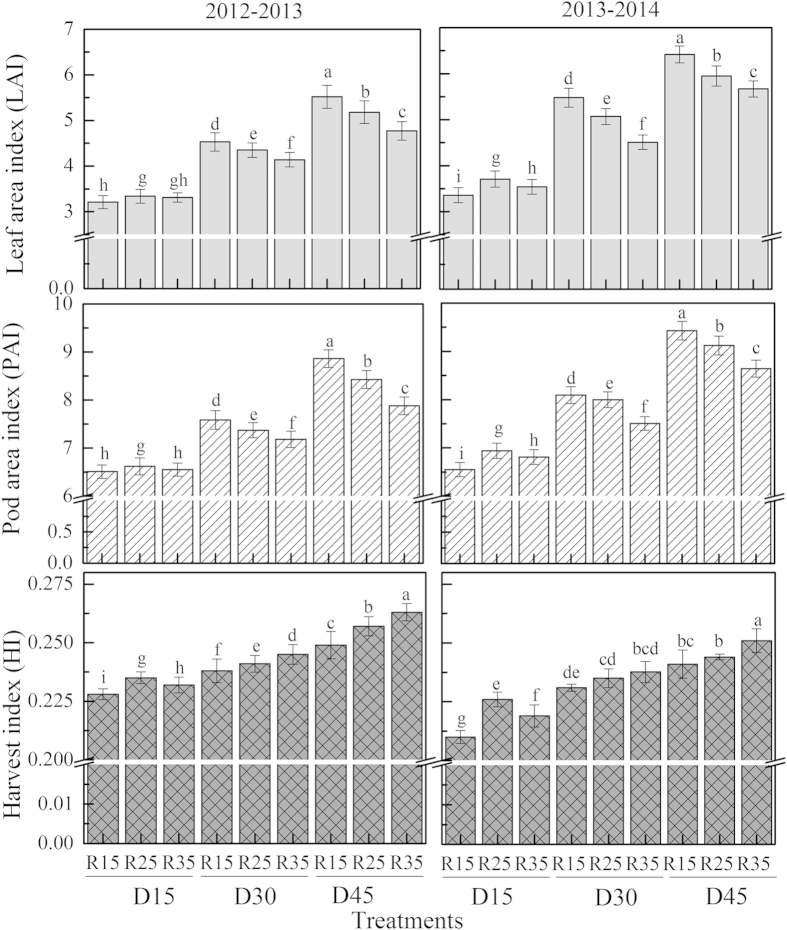
LAI, PAI and HI for direct-seedling winter rapeseed under different densities and row spacing arrangements during 2012–2014. D15, D30 and D45 mean densities of 15, 30 and 45 × 10^4^ plants ha^−1^; R15, R25 and R35 mean inter-row spacing of 15, 25 and 35 cm. Different letters denote significant difference at P < 0.05 by LSD.

The leaf area index (LAI), pod area index (PAI) and harvest index (HI) were all significantly affected by plant density and row spacing, but plant density had a greater impact on these variables (Fig. 1). The LAI and PAI increased significantly with increasing plant density. The effect of row spacing on the LAI and PAI was influenced by plant density. Under lower plant density (D15), the highest LAI and PAI were obtained in R25 followed by R35 and R15. Under higher plant densities (D30 and D45), the LAI and PAI both decreased with increasing row spacing. In all of the treatments, the combination of D45 plant density and R15 row spacing showed the highest LAI and PAI, whereas the combination of D15 plant density and R15 row spacing showed the lowest LAI and PAI in both growing seasons. A different trend was observed for the HI. In general, the HI increased significantly with increasing plant density and row spacing, with the highest and lowest values observed under D45R35 and D15R15, respectively.

### Light interception and radiation use efficiency (RUE)

**Figure 2:**
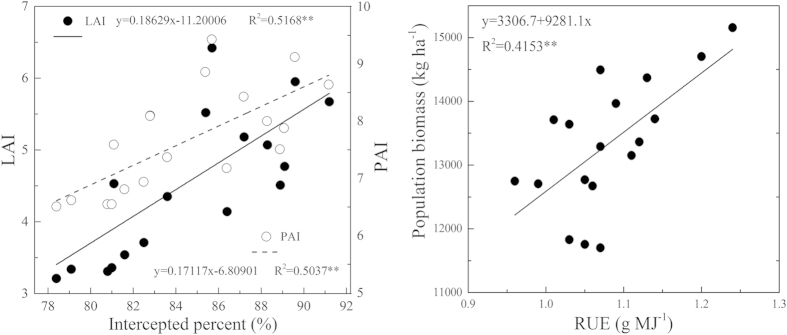
Relationships between (**a**) intercepted percent and LAI, PAI; (**b**) population biomass and RUE for direct-seedling winter rapeseed under different densities and row spacing arrangements (R^2^_0.05_ = 0.2191, R^2^_0.01_ = 0.3477).

The crop canopies intercepted approximately 80–90% of the incident radiation during the entire growing period. Generally, radiation interception increased with increasing plant densities and row spacing, reaching a peak of approximately 90% interception in 35 cm rows combined with 45 × 10^4^ plants ha^−1^. Under D15, the RUE increased with increasing row spacing, whereas under D30 and D45 conditions, a higher RUE was found in 15 cm rows. A similar trend was observed in above-ground biomass per m^2^ (Table 4). Further analyses revealed that percent interception was positively correlated with the LAI (*R*^*2*^ = 0.5168^**^) and PAI (*R*^*2*^ = 0.5037^**^), whereas population biomass was positively correlated with the RUE (*R*^*2*^ = 0.4153^**^) (Fig. 2).

### Mechanical harvesting properties

Overall, higher plant density significantly reduced plant height, snapping resistance, angle of lodging and culm lodging index, whereas the pod-shatter resistance increased at higher plant densities. Under higher plant densities (D30 and D45), the plant height, angle of lodging and culm lodging index clearly decreased, but the snapping resistance, pod-shatter resistance and seed moisture increased with increasing row spacing. The lowest plant height, angle of lodging and culm lodging index were observed under the D45R35 combination. Root biomass increased with row spacing under higher plant densities (D30 and D45). The maximum root biomass was observed under D15R25, whereas the minimum root biomass was found under D45R15. The root/shoot ratio was maximized under the highest plant density and the widest row spacing (D45R35) and minimized under D15R15. Under the lowest plant density (D15), the branching angle of the five lowest branches did not change with row spacing; however, it significantly decreased with increasing row spacing under higher plant densities (D30 and D45). In all treatments, D15R15 produced the lowest mechanical harvesting yield in both seasons. The mechanical harvesting yield increased significantly with plant density and row spacing. The highest mechanical harvesting seed yield with the lowest yield loss was obtained with the combination of R15 row spacing and D45 plant density. An analysis of variance revealed that mechanical harvesting properties were markedly affected by year, plant density and row spacing, with density having the greatest impact. Interactions between plant density and row spacing were significant for all of the evaluated mechanical harvesting properties (Table 5).

### Harvest loss rate

**Figure 3:**
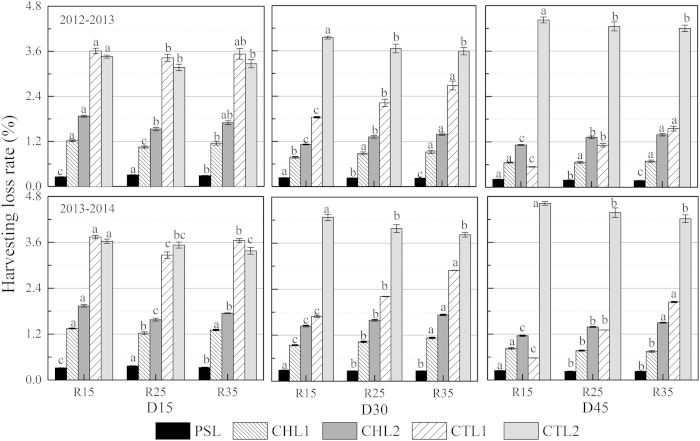
Loss rates of PSL (pod shatter loss pre-harvesting), CHL1 (shatter seed loss caused by combine header), CHL2 (branches loss caused by combine header), CTL1 (loss of unthreshed pods) and CTL2 (loss of shattered seeds mixed up with the straw) for direct-seedling winter rapeseed under different densities and row spacing arrangements during 2012–2014. D15, D30 and D45 mean densities of 15, 30 and 45 × 10^4^ plants ha^−1^; R15, R25 and R35 mean inter-row spacing of 15, 25 and 35 cm. Different letters denote significant difference at P < 0.05 by LSD.

Loss from cleaning and threshing (CTL) accounted for the largest proportion of yield loss, whereas the loss from natural pod shattering (PSL) accounted for the smallest proportion of yield loss. Plant density and row spacing had little impact on PSL and the loss from the combine header (CHL), whereas the loss rate of un-threshed pods in the straw (CTL1) and the loss rate of shattered seeds in the straw (CTL2) showed marked changes under different plant densities and row spacings. Increasing plant density significantly reduced the PSL, the loss rate of the seed shattered by mechanical harvesting (CHL1) and the loss rate of seeds on the branches remaining in each mechanical harvesting area (CHL2), whereas the CTL2 loss rate increased with increasing plant density. Under higher plant densities (D30 and D45), increasing row spacing reduced the PSL and CTL2 loss rates, whereas the CHL1, CHL2 and CTL1 loss rates increased (Fig. 3).

## Discussion

Plant densities of 15 × 10^4^ − 45 × 10^4^ plant ha^−1^ and row spacings of 15–35 cm have long been known to improve the seed yield of rapeseed in the Yangzi River region. However, rapeseed plant spacing requirements vary depending on the plant density, which has always been neglected by farmers in China. The recommended combination of appropriate density and row spacing for mechanical harvesting has not been reported. Consequently, research related to the combined effects of plant density and row spacing are urgently needed.

Yield differences were observed between the two growing seasons. The seed yield in 2013–2014 was higher than in 2012–2013. More sunshine and higher temperatures during the seedling and pod-filling stages, as well as during the entire growth period, in 2013–2014 compared with 2012–2013 were favorable for seedling and seed development (Table 1). In addition, more precipitation during the flowering to pod-filling stages in 2012–2013 resulted in more severe pod shattering^24^, which was unfavorable for production.

Different spatial arrangements produced by changes in row spacing can affect resource competition relationships at both the intra-specific and the inter-specific levels. Plants growing in rows that are too wide may not efficiently utilize natural resources such as light, water and nutrients, whereas growing in rows that are too narrow may result in severe inter- and intra-row spacing competition. Intra-specific competition has three effects: (i) density-dependent mortality, (ii) trade-offs between size and density, and (iii) alterations to population size structure^25^. The first two effects can be inferred by changes in average yield components, whereas the distribution of plant sizes within the crop provides information about the population structure. In the present study, plant mortality increased with reduced plant spacing as plant density and inter-row spacing increased (Table 2), indicating serious intra-specific competition under these conditions. The rapeseed plants adjusted to growing conditions and stand differences by changing the number of pods per plant, the number of seeds per pod and the 1000-seed weight (Table 3). There was a significant interaction between plant density and row spacing for yield and yield components, and plant density had a significantly greater influence than row spacing. Among the yield components, the number of pods per plant was most affected by plant density and row spacing, as reflected by the high coefficient of variation. Higher plant density and wider row spacing produced fewer pods per plant, resulting in a reduced yield per plant. The number of seeds per pod and the 1000-seed weight decreased with plant density, but increased with row spacing; this may have occurred because the rapeseed compensated for a loss in number of pods per plant with more seeds and higher seed weight, as has been previously reported^13^. Although wider rows produced more seeds and larger seeds, these were not sufficient to compensate for the loss in yield per plant caused by a decreased number of pods per plant when row spacing increased at the same plant density.

Under the conditions of this study, yield superiority was due primarily to the size of the plant population, where each plant was allowed a specific growing area, rather than the intra- or inter-row spacing individually. Compared with wider rows, narrower row spacing under higher plant density increased the distance between the plants within the row, resulting in a more equidistant planting pattern that is expected to delay the initiation of intra-specific competition as well as reduce intra-row competition^26^ while increasing early crop growth^27^. Leach *et al.* (1999) found that, in a series of multi-factorial experiments, the seed yield of winter oilseed rape increased with plant densities up to 50–60 × 10^4^ plants ha^−1^ 10. Besides, cultivar Huayouza 62 was planted by mechanical seeding with five planting densities (15 × 10^4^, 30 × 10^4^, 45 × 10^4^, 60 × 10^4^, and 75 × 10^4^ plant ha^–1^) in two growing seasons (2011–2012 and 2012–2013). Results showed that the direct seed rapeseed could obtain the highest yield under both densities of 30 × 10^4^ plants ha^−1^ and 45 × 10^4^ plants ha^−1^. Density higher than 45 × 10^4^ plants ha^−1^ results in strong competition, increases the potential for cooperation and also increases the difficulty for mechanical production^28^. As expected, the highest yield was observed under D45 in combination with R15, where the plant spacing and row spacing were similar, indicating that higher yields were associated with a more even plant distribution and a lower degree of intra-row competition. These findings are in agreement with Morrison *et al.* (1990)^13^, Argadi *et al.* (2003)^29^ and Karchi and Rudich (1966)^30^, who reported that uniformly distributed plant populations had significant advantages in yield over plant populations that were not uniformly distributed.

The combination of plant density and row spacing defines the spatial distribution of the plants, which affects canopy structure, light interception and radiation use efficiency and consequently, biomass production^31^. By narrowing the row width, a nearly optimum canopy display of leaves could be achieved resulting in greater seed yields than in wide rows^32^. In the present study, the population biomass showed a response to plant density and row spacing similar to yield. Variation in the population biomass could arise as a result of differences in radiation intercepted by the canopy, RUE and partitioning among different tissues^33^. Previous studies reported that during the period of most intensive accumulation of dry matter in the seeds, the proportion of assimilated carbon delivered by the photosynthetic tissues to the growing seeds was 37% for the leaves, 32% for the fruit walls, 31% for the stem and 1% for the seeds^34^, indicating that the leaves and the pods made the most important contributions to the yield of rapeseed. The potential increase in yield under D45R15 was closely related to the dense plant canopy, as seen in LAI and PAI (Fig. 1). The PAI was greater than the LAI, indicating that pods may be very important in providing photosynthates for their own development because they receive much higher radiation intensities than the leaves, although their photosynthetic rate was lower than that of the leaf^35^. Changes in the population yield under varying plant density and row spacing are affected not only by the rate of photosynthesis per unit of leaf area and the total area of photosynthetically active surfaces, but are also affected by the penetration of photosynthetically active light into the canopy because changes in row spacing and plant density could alter the timing of canopy closure by changing leaf area or pod area. Early canopy closure at higher plant density and narrower row spacing not only maximizes light interception between rows, but it evidently allowed more photosynthetically available radiation (PAR) to be transmitted between plants within rows. In addition, it also decreases evaporation from the soil surface and inhibits weed growth^36^. As expected, in this study, the percentage of intercepted radiation increased as plant density and row spacing increased, with the maximum interception being observed under D45R35 (Table 4). Significant positive correlations were observed between the LAI and percentage of intercepted light (*R*^*2*^ = 0.5168^**^), as well as between the PAI and percentage of intercepted light (*R*^*2*^ = 0.5037^**^) (Fig. 2). Allen and Morgan (1972) observed that the number of pods and the number of seeds per pod were positively correlated with the LAI at the onset of flowering^37^. This indicated that the difference in light interception related to LAI and PAI was one reason for yield changes under varying plant densities and row spacings. However, biomass production in rapeseed is more closely related to the utilization of solar radiation than to its interception because the RUE was significantly correlated with population biomass (Fig. 2). The influence of row spacing on the RUE was dependent on plant density. It had a response similar to the yield, with the maximum RUE being observed under narrow row spacing (15 cm) and under higher plant density (45 × 10^4^ plants ha^−1^) (Table 4). Overall, our values of RUE are lower than those of previous researchers as reported by Fletcher *et al.* (2012)^38^. There may be a genotypic difference among rapeseeds^39^ or lower air temperatures in winter could limit leaf photosynthesis^40^. The harvest index (HI) is one of the indices currently used to evaluate a crop’s partitioning efficiency. HI has been shown to improve with earlier maturity and to be negatively correlated with height and lodging score^23^, which is consistent with our results. HI clearly increased with plant density and row spacing (plant height and lodging were all reduced under this condition, as seen in Table 5), indicating that more photosynthate was distributed to the economic portions of the plant. Taken together, these results indicated that sowing rapeseed in narrow rows at higher plant density created a dense canopy and increased the light interception, RUE and distribution of photosynthetic production to the harvestable portion of the plant, leading to a yield increase in our study.

Stem lodging and pod shattering represent the most serious constraints to high yield during the mechanical harvesting of rapeseed. Experiments with natural and artificially induced lodging have shown that lodging-induced yield losses can range between 0 and 80%^41^, and stems lodged at 45° resulted in less yield loss than stems lodged at 80°^42^. Shattering can result in a yield loss of up to 50% by decreasing the overall dry matter production as well as all of the major yield components^43^. The combined effects of plant density and row spacing were studied here to find an optimum plant density and row spacing arrangement for the mechanical harvesting of rapeseed.

Because the plants lodged from the base of the stem, a weak stem or a shallow root system was presumed to be the cause of lodging. Most investigators have concluded that plots seeded at narrow row spacing and at high seeding rates produced plants with thinner stems that were less able to support the weight of the pods and the seeds and were more sensitive to lodging^44^. High plant density and narrow row spacing created a dense canopy where growing plants receive a different quality of light, enriched with far red (FR) and impoverished in red (R) radiation. This high FR/R ratio triggers many morphological changes in plant architecture, stimulating stem elongation, favoring apical dominance and reducing the stem diameter^45^. Such changes might make stems more susceptible to breakage before seeds reach physiological maturity. However, other results have suggested that the l5 cm row spacing produced significantly greater yields and lodged less frequently than the 30 cm row spacing at seeding rates of 1.5–12.0 kg ha^−1^ 13. We found that the lodging index and the angle of lodging both decreased at higher plant density and wider row spacing, with the minimum values being observed under D45R35 (Table 5). The modern hybrid morphological traits may have helped to mitigate stem lodging at high plant population densities. Plant height is important when considering harvest equipment and has been targeted in efforts to improve lodging resistance^46^. Lodging resistance was greatly influenced by plant density and row spacing and was higher in wide-row compared with narrow-row treatments in soybeans [Glycine max (L.) Merr.]^35^. In contrast, in our research, there was decreased plant height of rapeseed under higher plant density and wider row spacing, possibly because the field was rain-fed, without any irrigation during the entire development stage, and thus intra-specific competition existed for water, which limited stem elongation. At sites with adequate soil moisture, mature plant heights increased as seeding rates increased, whereas at moisture-deficient sites, plant height was not significantly affected by increased seeding rates^47^. In addition, closer spacing within the row resulted in partial shading at earlier growth stages, and partial shading can change the light quality in the canopy, triggering plants to develop longer stem internodes^48^. Therefore, another reason for decreased plant height in this study might be that higher plant density and appropriate row spacing lowered the number of upright branches, and the pod numbers may have reduced the selective absorption of red light by the upper canopy, improving light quality and decreasing stem elongation. Higher plant density reduced the root biomass, but an irregular trend in root biomass was affected by row spacing in both seasons. However, the root-shoot ratio clearly increased as plant density and row spacing increased (Table 5), suggesting relatively better root growth under these conditions, which enhanced water and nutrient uptake. The short plant height and increased root-shoot ratio were probably instrumental in maintaining the plant’s gravitational center close to the ground, a key feature in sustaining the canopy weight until harvesting. Similar results were reported by Sangoi *et al.*^49^. Altering the pattern of plant spacing by changing plant density and row spacing also had impact on pod-shatter resistance. The increased HI improved the dry matter accumulation of the pod wall under the highest plant density and widest row spacing, and the same trend was observed for pod-shatter resistance (Table 5). In a previous study, we found that there was a significant correlation between pod-shatter resistance and pod dry weight^50^. Consequently, the increased biomass led to a more compact pod wall, which improved the ability of the pod to resist shattering.

Changes in agronomic traits, especially in lodging, pod shattering and canopy architecture, could affect the mechanical harvesting yield. At low seeding rates, a large proportion of the total yield is produced on the branches, and as the seeding rate increases, the yield from the branches decreases^13^ indicating that branch growth is inhibited as plant density increased. Similar results were observed in the present study. The angle of the five lower branches significantly decreased with increasing row spacing under higher plant densities (D30 and D45) (Table 5). Rapeseed, with a greater capacity for altering branch display angles, was well-suited for efficient light interception and mechanical harvesting operations in narrow rows under higher density, which ultimately reduced mechanical harvesting yield loss. The highest mechanical seed yield was obtained for the combination of 15.0 cm row spacing and 45 × 10^4^ plants ha^−1^ plant density without a serious yield loss resulting from lodging and pod shattering, with decreased branching angles (Table 5) and with a higher theoretical seed yield. Similar results were reported by Bilgili *et al.* (2003)^44^.

Previous studies have shown that seed loss of rapeseed decreased by 5%–10% under direct harvesting with a combine harvester^43^, which was slightly lower than our results. The yield loss in the present study was between 7.0% and 11.0% (Table 5). Total yield loss consisted of loss from natural pod shattering, loss from the combine header and loss from cleaning and threshing. An optimal level of moisture was present in the seeds during harvesting with the combine harvester^24^,^51^, decreasing seed loss and damage. It was shown that a seed moisture content of 15%–20% could limit seed loss to within 11% (Table 5). The rate of PSL and CHL loss was largely influenced by weather conditions and combine header operation, respectively, while the loss from CTL was primarily affected by agronomic practices. According to the loss rates in the current study, the minimum seed loss was associated with PSL and the maximum loss was observed in CTL, which was caused by cleaning and threshing. This indicates that optimum plant density combined with appropriate row spacing helped to maintain the uniformity of the canopy and could significantly reduce yield loss caused by mechanical harvesting. Among the different types of loss rates, CTL1 was most significantly affected by plant density and row spacing (Fig. 3). Under the combination of plant density and row spacing, the minimum yield loss under D45R15 was primarily due to the low CTL1 loss rate. Plants at low population densities produce more branches that carry fertile pods, thus prolonging the seed development phase. This results in a range of seed maturities at harvest, which may affect seed quality, increase the risk of seed loss through pod shatter and impede harvesting. Decreased yield loss was observed with increasing plant density and narrowing row spacing because of an improvement in resistance to stem lodging and pod shattering, decreased branching angles and the presence of fewer pod-bearing branches that produced more synchronous pods and seed development and resulted in more uniform seed maturation, which facilitated harvesting^10^.

## Conclusion

Uniform row spacing and plant spacing (a combination of 15.0 cm row spacing and 45 × 10^4^ plants ha^−1^ plant density) could improve the seed yield and mechanical harvesting efficiency for direct-seeded rapeseed. This combination produced a more compact canopy architecture, leading to a higher LAI and PAI, enhanced solar radiation and RUE, and subsequently, higher crop biomass. All of these factors contributed to a high yield. In addition, this combination also led to shorter plants, increased root-shoot ratios, reduced branching angles and promoted greater resistance to stem lodging and pod shattering. These various agronomic traits facilitated mechanical harvesting. Overall, these results suggest that producers should be encouraged to seed rapeseed in narrowly-spaced rows with high plant density. The combination of 15.0 cm row spacing and 45 × 10^4^ plants ha^−1^ plant density could be considered an optimum cultivation practice for mechanized production of direct-seeded rapeseed in Central China.

## Methods

### Experimental site

A replicated field experiment was conducted during two seasons (2012–2013 and 2013–2014) at the Huazhong Agricultural University Experimental Farm (30°28′12″N, 114°21′05″E) in Wuhan, China. The previous crop, rice, was harvested in September. The initial soil status (0–20 cm) of the field in terms of available nitrogen (N), phosphorus (P) and potassium (K) concentrations was as follows: 101.26 mg kg^–1^, 13.84 mg kg^–1^ and 146.28 mg kg^–1^, respectively, in 2012–2013 and 103.63 mg kg^–1^, 14.47 mg kg^–1^ and 150.38 mg kg^–1^, respectively, in 2013–2014. Table 1 shows the rainfall and temperature during the two growing seasons; these data were provided by the National Meteorological Information Center of the China Meteorological Administration.

### Experimental design

**Figure 4:**
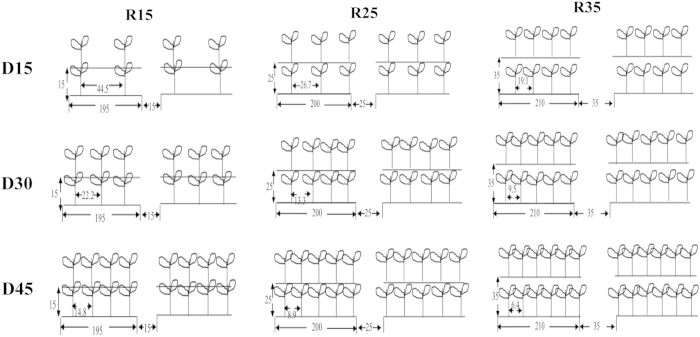
The schematic diagram for direct-seedling winter rapeseed under different densities and row spacing arrangements during 2012–2014 (unit: cm). D15, D30 and D45 mean densities of 15, 30 and 45 × 10^4^ plants ha^−1^; R15, R25 and R35 mean inter-row spacing of 15, 25 and 35 cm.

Huayouza 62, a popular winter rapeseed hybrid cultivar in Central China, was used in this study. The experiment had a split-plot design with three plant densities (D1, 15 × 10^4^ plant ha^−1^; D2, 30 × 10^4^ plant ha^−1^; D3, 45 × 10^4^ plant ha^−1^) as the primary plots and three row spacings (R1, 15 cm; R2, 25 cm; R3, 35 cm) as the split plots. The row arrangements are illustrated in Fig. 4; inter-row spacings and plant densities varied in each plot (Table 2). The plant densities of 15 × 10^4^–45 × 10^4^ plant ha^−1^ and row spacings from 15–35 cm used in the study are widely used for rapeseed production in the Yangzi River region. Each treatment was performed in three replicate plots.

Rapeseed was manually sown on the 23^rd^ of September and the 26^th^ of September in 2012 and 2013, respectively. The seed density was evaluated directly after seedling emergence and adjusted for precise planting density at the five-leaf growth stage for all plots. Before sowing, 900 kg ha^−1^ of N, P and K compound fertilizer (N:P:K = 15:15:15) and 7.5 kg ha^−1^ of borax (for boro) were applied as a basal fertilizer. During the wintering stage, 135 kg ha^−1^ nitrogen from urea was applied. Pest, disease and weed control were performed according to local management practices.

### LAI, PAI and RUE

The Leaf area was measured on 20 plants in each plot at the bolting stage using a leaf area-meter (Li-3100c, Li-COR Inc., USA). The leaf area index (LAI) was defined as the ratio of total one-sided leaf area to ground surface area^52^. The pod area was measured on 20 plants in each plot 30 days after the end of flowering according to the formula: 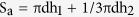 (where h_1_ = 0.8 H, h_2_ = 0.2 H, H is pod length and d is pod width)^53^. The pod area index (PAI) was defined as the ratio of total pod area to ground surface area. Canopy radiation interception was measured using a SunScan Canopy Analysis System (Delta-T Devices Ltd., UK) at the seedling, wintering, bolting, flowering, pod-filling and maturity stages as suggested by Wang *et al.* (2015), with some modification^54^. Canopy radiation interception was calculated as [100 × (incoming radiation intensity– radiation intensity inside canopy)/incoming radiation intensity]. Intercepted radiation was calculated as [1/2 × (canopy radiation interception at the beginning of the growth period + canopy light interception at the end of the growth period) × accumulated incoming radiation during the growth period]^55^. The radiation use efficiency (RUE) was calculated as the ratio of above-ground total dry weight at maturity to intercepted radiation during the entire growing season^52^.

### Measurement of lodging behavior

The lodging degree, snapping resistance, culm lodging index and pod-shatter resistance were measured as previously reported by Kuai *et al.* (2015)^50^.


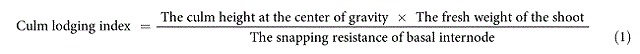



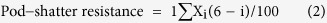


where i is the frequency of agitation and 1 ≤ i ≤ 5 and X_i_ is the number of broken pods.

After the random impact tests, the dry weight of the pod was recorded.

### Assessment of manually harvesting seed yield and yield components

Plots were harvested when approximately 2/3 of the seed was brown. Ten plants from each plot were randomly sampled and slowly uprooted and the taproot and large lateral roots were retained. Next, the yield components and seed yield per plant were determined. On each plant, the following measurements and observations were made: the plant height (cm), the angle of the lowest 5 branches, pods per plant, seeds per pod and the 1000-seed-weight (g). Then, the remaining plants of 8 m in each plot were manually harvested to measure the manually harvesting seed yield (Y_mn_, kg ha^−1^). The heights of the plants were measured from the cotyledonary node to the ligule of the uppermost fully expanded leaf, and the average height was calculated from all plants measured. Plant tissue samples were separated from the cotyledonary node into roots and aboveground tissues. After the fresh weight was determined, the roots and the aboveground tissues were dried in an oven for 30 min at 105 °C to deactivate enzymes and then dried at 70 °C until a constant weight was reached for dry weight determination.

### Assessment of yield loss and mechanical harvesting yield

**Figure 5:**
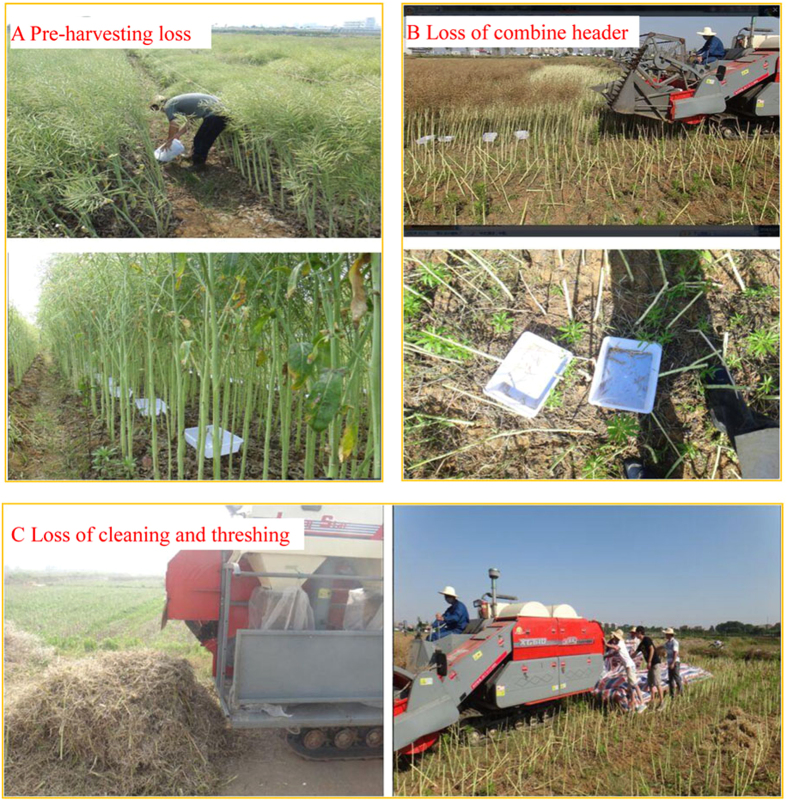
Schematic operation for determination yield losses in the study. (**A**) loss of naturally pod shattering pre-harvesting; (**B**) loss of combine header; (**C**) loss of cleaning and threshing.

A combine harvester (4LL-2.0D, Huzhou, China) was used for mechanical harvesting when the seed moisture of pods from the main inflorescences was 12%–13%. Each plot was harvested over a length of 40 m, maintaining a 0.4 m cutting height. Three sources of losses were assessed as shown in Fig. 5.

A. Loss from natural pod shattering (PSL): The pre-harvest losses due to natural dehiscence and environmental conditions, e.g., rainfall, wind, or birds. Because the direct collection of seeds that have fallen to the soil surface is impracticable, 10 plastic trays (25 cm × 15 cm × 5 cm) were positioned inside each plot, as described below, when approximately 2/3 of the seeds in the field were brown (the manual harvesting date)^43^,^56^. These trays were removed just prior to harvesting and the seed was weighed (W_PSL_). The pre-harvesting loss rate (PSL) (%) = W_PSL_/Y_mn_ × 100.

B. Loss from combine header (CHL): The shattered seed loss and branch loss attributable to the combine header. The measurement of shattered seed loss during mechanical harvesting was conducted by placing 10 trays on the ground in each plot. The trays were placed 8 m before the end of the plot (the plot was 40 m in length) to allow for the combine to pass over them while operating (threshing, separating and cleaning). The distance before the trays (32 m) was also sufficient to allow the combine to transit above the trays in full operation. The weight of the seed shattered by mechanical harvesting was recorded. The loss rate of these seeds was represented by (CHL1) (%) = W_CHL1_/Y_mn_ × 100. The branches remaining in each mechanical harvesting area (40 m in length) after harvest were collected, and the seeds on these branches were weighed. The loss rate of seeds on these branches was represented by (CHL2) (%) = W _CHL2_/Y_mn_ × 100.

C. Loss from cleaning and threshing (CTL). The rear of the harvester was covered with a nylon membrane 3 m in width; the straw was collected for 20 m along each plot. The seeds of un-threshed pods and shattered seeds in the straw were collected and weighed separately. The loss rate of un-threshed pods in the straw was represented by (CTL1) (%) = W_CTL1_/Y_mn_ × 100. The loss rate of shattered seeds in the straw was represented by (CTL2) (%) = W_CTL2_/Y_mn_ × 100. All seeds freely falling from the frame of the harvester were collected and weighed to determine the mechanical harvesting yield.

The total loss rate (%) was determined as (Y_mn_ −Y_m_)/Y_mn_ × 100, where Y_m_ = mechanical harvesting seed yield and Y_mn_ = manually harvesting seed yield.

### Statistics analysis

Two-way analysis of variance (ANOVA) was performed using Duncan’s multiple range test. Significant differences in means between the treatments were compared by the protected least significant difference (LSD) procedure at P < 0.05. ANOVA and the LSD test were conducted using the SPSS 17.0 software program. Figures were prepared using the Origin 9.0 software program.

## Additional Information

**How to cite this article**: Kuai, J. *et al.* The yield of mechanically harvested rapeseed (*Brassica napus L.*) can be increased by optimum plant density and row spacing. *Sci. Rep.* **5**, 18835; doi: 10.1038/srep18835 (2015).

## Figures and Tables

**Table 1:** Meteorological conditions during the growing seasons of oilseed rape in 2012–2014.

Year	Meteorological factors	Seedling to wintering stage	Wintering to flowering stage	Flowering to pod filling stage	The whole growth stages
2012–2013	Monthly Precipitation (mm)	78.3	77.4	169.5	885.2
	Monthly sunshine duration (h)	132.6	105.3	154.7	1188.4
	Accumulated temperature (°C)	1180.9	315.5	943.1	2439.5
2013–2014	Monthly Precipitation (mm)	64.4	82.7	152.4	791.9
	Monthly sunshine duration (h)	174.3	87.9	147.6	1342.3
	Accumulated temperature (°C)	1437.1	261.2	1162.2	2860.5

**Table 2:** The plant spacing and mature plant rate under different densities and row spacing arrangements during 2012–2014.

Density	Row Spacing	Plant spacing (cm)	Maturity rate (%)	
			2012–2013	2013–2014
D15	R15	44.5	93.07c	90.09b
	R25	26.7	98.10a	96.84a
	R35	19.1	96.27b	92.22b
D30	R15	22.2	90.67d	86.55c
	R25	13.3	84.99e	81.12d
	R35	9.5	80.03f	74.62e
D45	R15	14.8	85.20e	78.48d
	R25	8.9	78.63f	71.70e
	R35	6.4	70.30g	64.00f

Values followed by different letters within the same column are significantly different according to the least significant difference (LSD) test (P < 0.05); Each data represents the mean of three replications; D15, D30 and D45 mean densities of 15, 30 and 45 × 10^4^ plants ha^−1^; R15, R25 and R35 mean inter-row spacing of 15, 25 and 35 cm.

**Table 3:** Seed yield and yield components of winter rapeseed under different densities and row spacing arrangements during 2012–2014.

Year	Density	Row Spacing	Biomass per plant (g)	Population biomass (kg ha^−1^)	Pods per plant	Seeds per pod	1000-seed weight (g)	Seed yield per plant (g)	Manually seed yield (kg ha^−1^)
2012–2013	D15	R15	84.74a	11829.7e	303.4a	18.3bc	3.48a	19.31a	2616.0h
		R25	79.53b	11702.8e	290.8b	18.9a	3.40b	18.68b	2689.5g
		R35	81.42b	11757.0e	295.1ab	18.5b	3.46a	18.89ab	2658.0g
	D30	R15	50.46c	13725.1b	201.9c	17.7d	3.36cd	12.00d	3190.5d
		R25	51.58c	13151.9c	204.3c	18.0cd	3.38bc	12.43cd	3088.5e
		R35	52.78c	12672.5d	208.4c	18.3b	3.39b	12.93c	2997.0f
	D45	R15	38.35d	14703.4a	181.4d	16.1c	3.27f	9.55f	3543.0a
		R25	38.75d	13711.7b	183.5d	16.4f	3.31e	9.96f	3387.0b
		R35	40.30d	12748.9d	186.6d	17.0e	3.34d	10.59e	3243.0c
	Coefficient of variation (CV%)		33.20	8.05	22.76	5.52	1.98	29.07	10.95
	Mean		57.55	12889.2	228.4	17.7	3.38	13.82	3046.5
2013–2014	D15	R15	98.89a	13363.9cd	324.7a	19.3bc	3.33a	20.78a	2721.0h
		R25	87.91c	12770.2d	307.0c	20.1a	3.22c	19.86b	2814.0g
		R35	91.86b	12706.9d	312.9b	19.6b	3.28b	20.10b	2746.5h
	D30	R15	55.34e	14369.0b	213.8f	18.8d	3.18def	12.78e	3220.5d
		R25	57.39de	13965.9bc	222.5e	19.0cd	3.19cde	13.48d	3183.0e
		R35	59.37d	13290.6cd	231.4d	19.1cd	3.21cd	14.10c	3094.5f
	D45	R15	42.92g	15156.9a	196.8h	16.9f	3.11g	10.34h	3612.0a
		R25	44.92fg	14493.4b	202.3g	17.2f	3.15f	10.96g	3465.0b
		R35	47.36f	13640.4c	211.9f	17.7e	3.17ef	11.88f	3340.5c
	CV%		33.39	5.96	21.07	5.95	2.08	27.92	10.19
	Mean		65.11	13750.8	247.0	18.6	3.20	14.92	3133.5
Analysis of variance	Year (Y)		125.3^**^	32.4^*^	41.4^*^	180.9^**^	477.3^**^	46.3^*^	1685.0^**^
	Density (D)		4685.3^**^	136.3^**^	4473.9^**^	305.4^**^	269.1^**^	5291.5^**^	4650.4^**^
	Row spacing (R)		10.0^**^	76.1^**^	9.5^**^	51.7^**^	27.6^**^	23.1^**^	243.5^**^
	Y × D		18.9^**^	3.5NS	0.4NS	1.6NS	1.5NS	1.6NS	2.5NS
	Y × R		0.9NS	0.1NS	2.5NS	1.4NS	1.5NS	0.6NS	3.5^*^
	D × R		22.4^**^	11.3^**^	15.0^**^	16.5^**^	45.2^**^	19.6^**^	116.1^**^
	Y × D × R		3.0^*^	1.8NS	1.3NS	1.0NS	0.8NS	0.8NS	2.4NS

Values followed by different letters within the same column are significantly different according to the least significant difference (LSD) test (P < 0.05); Each data represents the mean of three replications.

NS means not significant; *and **means significant differences at 0.05 and 0.01 probability levels, respectively; D15, D30 and D45 mean densities of 15, 30 and 45 × 10^4^ plants ha^−1^; R15, R25 and R35 mean inter-row spacing of 15, 25 and 35 cm.

**Table 4:** Radiation use efficiency and its related parameters of winter rapeseed under different densities and row spacing arrangements during 2012–2014.

Year	Density	Row Spacing	Incident radiation (MJ m^−2^)	Intercepted radiation (MJ m^−2^)	Intercepted percent (%)	Total aboveground biomass (g m^−2^)	Radiation use efficiency (g MJ^−1^)
2012–2013	D15	R15	1312.5	1030.0cd	78.5f	1064.1f	1.03f
		R25	1218.4	964.7g	79.2f	1029.4h	1.07d
		R35	1292.1	996.2f	80.6e	1048.4g	1.05e
	D30	R15	1266.9	1027.0cd	81.1e	1175.6b	1.15b
		R25	1238.3	1009.1e	84.1d	1123.7d	1.11c
		R35	1226.7	1019.7de	86.5b	1079.1e	1.06de
	D45	R15	1208.9	1032.5c	85.4c	1238.7a	1.20a
		R25	1312.6	1138.0a	87.2b	1149.8c	1.01g
		R35	1243.3	1108.3b	89.1a	1062.7f	0.96h
	Mean		1257.7	1036.2	83.5	1107.9	1.07
2013–2014	D15	R15	1337.7	951.6h	82.7g	1062.1h	1.12c
		R25	1389.5	1126.6b	83.9f	1186.1d	1.05f
		R35	1353.7	1125.6b	83.2g	1110.4g	0.99h
	D30	R15	1280.9	986.3g	84.2f	1115.0f	1.13b
		R25	1302.8	1105.5c	89.7d	1208.2b	1.09d
		R35	1259.5	1089.7d	90.4c	1168.3e	1.07e
	D45	R15	1266.5	1014.6f	87.1e	1257.3a	1.24a
		R25	1358.3	1032.6e	91.0b	1107.9g	1.07e
		R35	1284.4	1160.3a	92.7a	1199.4c	1.03g
	Mean		1314.8	1065.9	87.2	1157.2	1.09

Values followed by different letters within the same column are significantly different according to the least significant difference (LSD) test (P < 0.05); Each data represents the mean of three replications.

D15, D30 and D45 mean densities of 15, 30 and 45 × 10^4^ plants ha^−1^; R15, R25 and R35 mean inter-row spacing of 15, 25 and 35 cm.

**Table 5:** Mechanical harvesting yield and mechanical properties of winter rapeseed under different densities and row spacing arrangements during 2012–2014.

Year	Density	Row spacing	Plant height (cm)	Root biomass (g)	Root/Shoot	Branching angle (°)			Snapping resistance (N)	Angle of lodging (°)	Culm lodging Index (g.cm N^−1^)	Pod shatter resistance	Seed moisture (%)	Pod wall biomass (g)	Mechanical harvesting yield (kg ha^−1^)	Total yield loss (%)
						the 1st lowest branch	the 3rd lowest branch	the 5th lowest branch								
2012–2013	D15	R15	164.6bc	8.56b	0.101e	44.65a	42.65a	36.59a	181.1a	23.8b	103.4a	0.029f	16.10a	0.036g	2343.5i	10.42a
		R25	167.9a	9.62a	0.121d	45.63a	42.71a	36.52a	170.4b	25.9a	104.1a	0.023i	15.94a	0.040f	2434.0g	9.49c
		R35	166.3ab	8.86b	0.109e	44.44a	42.13a	36.21a	174.9b	24.3b	103.9a	0.025h	15.99a	0.042ef	2394.0h	9.94b
	D30	R15	164.1bc	7.27d	0.144c	44.38a	41.78a	35.06b	129.4e	19.4c	89.3b	0.028g	14.61bc	0.043de	2927.5d	8.24ef
		R25	162.3c	7.53cd	0.146c	42.50b	40.51b	34.48bc	136.9d	18.4d	87.1b	0.032d	14.79b	0.044cd	2825.0e	8.53e
		R35	159.2d	7.86c	0.149c	41.33bc	39.23cd	33.29cd	148.2c	15.9e	84.2c	0.034c	14.90b	0.044cd	2725.5f	9.05d
	D45	R15	157.3d	6.06f	0.158b	41.56bc	39.41c	33.34cd	118.8f	15.4e	76.8d	0.031e	13.89d	0.045bc	3297.0a	6.94h
		R25	154.1e	6.28f	0.162ab	40.45c	38.37d	32.48d	123.7ef	7.3f	73.1e	0.038b	14.10cd	0.046ab	3132.0b	7.53g
		R35	150.9f	6.75e	0.168a	38.08d	36.17e	30.28e	127.8e	3.5g	72.3e	0.042a	14.32bcd	0.048a	2983.5c	8.01f
	Mean		160.7	7.64	0.14	42.56	40.33	34.25	145.7	17.1	88.2	0.031	14.96	14.96	14.96	8.68
2013–2014	D15	R15	177.1bc	11.17b	0.113g	45.54a	42.90a	36.44a	212.2a	26.0b	105.9b	0.033f	17.88a	0.039ef	2422.5i	10.98a
		R25	182.6a	11.52a	0.131e	44.92a	42.80a	36.24a	180.9c	27.2a	111.2a	0.027h	17.31bc	0.036f	2533.5g	9.98c
		R35	180.3ab	11.3ab	0.123f	45.23a	42.90a	36.37a	196.1b	26.5b	107.3b	0.031g	17.45ab	0.039ef	2460.5h	10.42b
	D30	R15	175.7cd	8.84e	0.160d	45.39a	42.35ab	35.76ab	141.1f	18.8c	93.7c	0.035e	16.12de	0.040de	2942.5d	8.63e
		R25	174.6cd	9.41d	0.164cd	43.43b	41.51bc	35.39bc	148.2e	18.0d	91.8c	0.038cd	16.38d	0.044bc	2894.5e	9.08d
		R35	172.1de	9.91c	0.167bcd	43.01b	40.99c	34.76c	165.6d	15.0e	88.2d	0.039c	16.90c	0.045b	2790.0f	9.84c
	D45	R15	171.5de	7.34h	0.171abc	43.12b	41.10c	35.04bc	124.7h	13.5e	83.2e	0.037d	15.26f	0.042cd	3343.5a	7.44g
		R25	170.1e	7.77g	0.173ab	41.28c	39.36d	33.38d	130.9g	7.2f	82.2ef	0.041b	15.74e	0.046b	3185.0b	8.08f
		R35	169.9e	8.29f	0.175a	39.70d	37.91e	32.45e	137.8f	4.8g	80.4f	0.044a	16.05de	0.050a	3049.0c	8.74e
	Mean		174.9	9.56	0.153	43.51	41.31	35.09	159.7	17.4h	93.8	0.036	16.57	16.57	2846.8	9.24
Analysis of variance	Year (Y)	1058.00^**^	449.20^**^	273.27^**^	25.22^**^	50.91^**^	42.15^**^	102.20^**^	11.40NS	198.40^**^	130.50^**^	695.70^**^	695.70^**^	764.01^**^	3162.02^**^	
	Density (D)		163.60^**^	704.70^**^	515.71^**^	177.93^**^	279.26^**^	253.29^**^	1386.40^**^	10092.60^**^	3069.30^**^	1695.70^**^	203.70^**^	203.70^**^	5080.48^**^	798.69^**^
	Row spacing (R)		5.76^**^	62.80^**^	32.84^**^	42.18^**^	57.71^**^	43.69^**^	42.90^**^	785.20^**^	13.60^**^	150.10^**^	5.70^**^	5.70^**^	315.86^**^	75.74^**^
	Y × D		6.10^*^	14.20^**^	2.14NS	2.79NS	5.58^**^	14.52^**^	16.90^**^	65.50^**^	16.40^**^	19.00^**^	0.40NS	0.40NS	2.76NS	0.27NS
	Y × R		1.70NS	2.50NS	0.03NS	2.61NS	2.66NS	2.98NS	5.90^**^	8.80^**^	2.80NS	6.50^**^	0.70NS	0.70NS	2.75NS	1.67NS
	D × R		6.30^**^	15.00^**^	12.86^**^	9.53^**^	13.06^**^	11.41^**^	50.20^**^	443.50^**^	7.70^**^	176.80^**^	5.40^**^	5.40^**^	160.47^**^	66.08^**^
	Y × D × R		0.50NS	4.10^*^	0.82NS	0.47NS	0.63NS	0.58NS	5.40^**^	13.60^**^	0.80NS	8.60^**^	1.20NS	1.20NS	1.66NS	0.92NS

Values followed by different letters within the same column are significantly different according to the least significant difference (LSD) test (P < 0.05); Each data represents the mean of three replications. NS means not significant; *and **means significant differences at 0.05 and 0.01 probability levels, respectively; D15, D30 and D45 mean densities of 15, 30 and 45 × 10^4^ plants ha^−1^; R15, R25 and R35 mean inter-row spacing of 15, 25 and 35 cm.
